# Desulfinative Cross-Coupling as a Method to Overcome Problematic Suzuki–Miyaura Reactions of Pharmaceutically Relevant Heteroaromatic Boronates

**DOI:** 10.1021/acsmedchemlett.5c00327

**Published:** 2025-07-23

**Authors:** David C. Blakemore, Andre Shavnya, Michael C. Willis

**Affiliations:** † Medicine Design, Pfizer Inc., Groton, Connecticut 06340, United States; ‡ Department of Chemistry, 6396 University of Oxford, Oxford OX1 3TA, United Kingdom

**Keywords:** pyridine, sulfinate, sulfone, cross-coupling, heterocycle, desulfinative, parallel synthesis

## Abstract

The well documented difficulties associated with direct (hetero)­arylation of *aza*-aromatics (e.g., azines) at the α-position to nitrogen led to a collaborative project between the Willis group at Oxford and the Medicine Design department at Pfizer with the aim of addressing this challenge. The result of this collaboration has been a series of reports detailing the development of 2-*aza*-aryl sulfinates, as well as related 2-*aza*-aryl sulfone derivatives, as efficient nucleophilic reagents in palladium-catalyzed coupling reactions with (hetero)­aryl halides. The developed chemistry is routinely used in the medicinal chemistry laboratories at Pfizer, and the patent literature now contains many examples of these methods being embraced across the pharmaceutical industry. Hundreds of pyridyl (and related heterocyclic) sulfinates are now commercially available from multiple vendors. In this microperspective we discuss the development and evolution of these methods and highlight subsequent applications.

## Graphical abstract

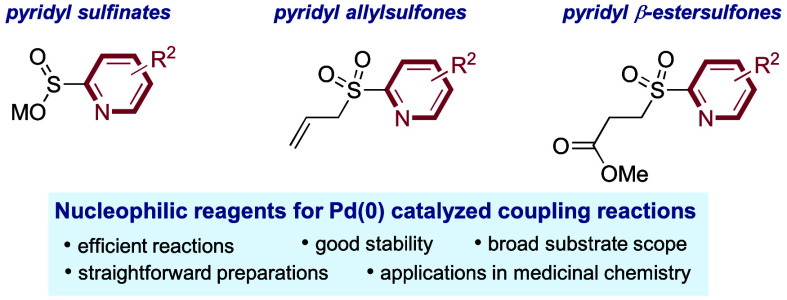

6-Membered *aza-*aromatic rings (pyridines, pyrimidines, pyridazines, pyrazines, triazines, and others) linked to further (hetero)­aromatics are a common feature in multiple medicines, with the 2-*aza*-aryl linkage being rather common (Figure 1).^1–5^ As well as being present in existing active pharmaceutical ingredients (APIs), this motif continues to be in demand in current medicinal chemistry programs. The logical approach to prepare these linked (hetero)­aromatics is to invoke transition metal catalyzed cross-coupling, with Pd-mediated reactions being most common. However, the use of 2-*aza-*aryl boronic acids and related boron reagents, needed to execute Suzuki-Miyaura couplings,^6,7^ is particularly challenging.^8,9^ The propensity of the required 2-boron reagents to undergo proto-deboronation is well documented,^10,11^ and is the cause of the instability of these reagents and their corresponding poor performance in the associated coupling reactions. These features have collectively become known as the “2-pyridyl problem”.^12,13^ Reported mitigations include the use of various boronate reagents^14–18^ or Lewis acidic additives,^19,20^ as well as a number of nonboron based approaches.^13,21–24^ However, in practical industrial applications many of these methods suffered from poor generality and/or the requirement for extensive substrate specific optimization; thus, this problem remained critical to resolve.

**Figure 1:**
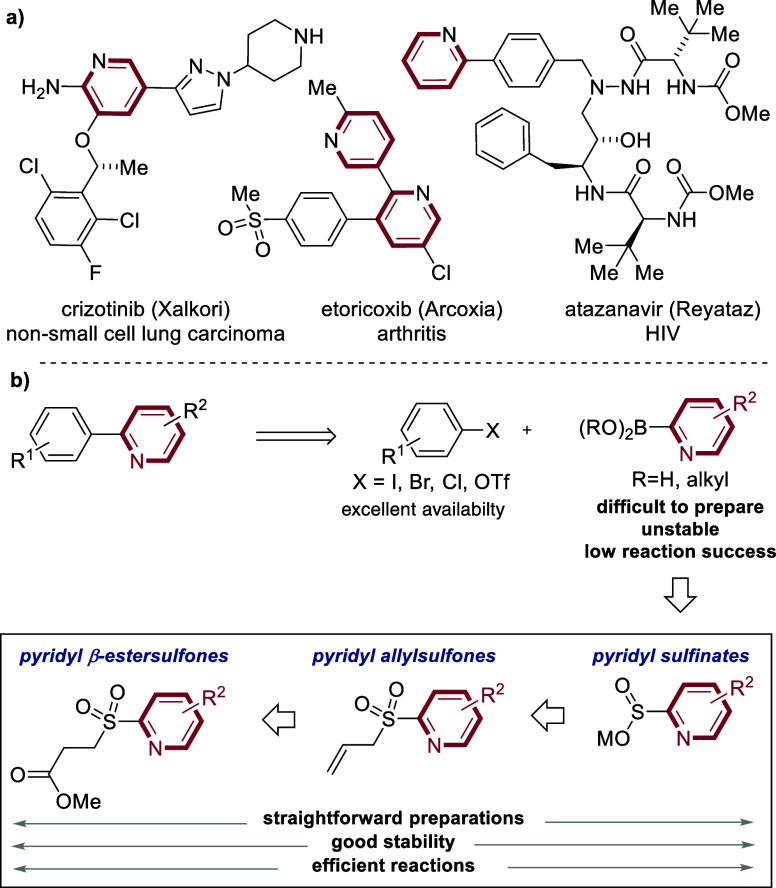
a) Pyridines in medicinal chemistry. b) 2-Pyridyl sulfinates, pyridyl allylsulfones, and pyridyl β-estersulfones as replacements for pyridyl 2-boronates.

In 2017 our laboratories initiated a collaborative program to achieve the efficient synthesis of 2-aryl- and 2-heteroarylpyridines. Initially, we set a series of aims: (i) the chemistry should use stable and easy to prepare coupling partners; (ii) it should deliver efficient reactions with both aryl bromide and chloride substrates; (iii) the ability to prepare the most challenging products, namely linked pyridine-heterocyclic molecules, should be possible; and (iv) ideally, there would be tolerance to the use of a single catalyst for all of the various coupling combinations, involving both aryl and heteroaryl substrates. The starting point for our investigations was the observation of the formation of aryl–aryl coupling side-products in studies on the palladium-catalyzed insertion of sulfur dioxide with aryl halides.^25,26^ Importantly, these side products were more pronounced with heteroaromatic substrates. These observations, combined with the existing literature on desulfinative coupling reactions of carbocyclic sulfinates,^27–31^ prompted us to explore 2-pyridyl sulfinates as nucleophilic coupling partners.

Our early work established that pyridine 2-sulfinates were excellent nucleophilic coupling partners in palladium-catalyzed reactions with (hetero)­aryl halides. Our first iteration of these reactions employed PCy_3_-derived palladium catalysts,^32^ and provided the coupled products in high yields. Scheme 1a shows selected examples, and as can be seen, the chemistry was effective for a range of substituted pyridyl sulfinates, and both aryl chlorides and bromides could be used as coupling partners. Heteroaryl halides were also good reaction partners, allowing the synthesis of a range of linked pyridyl-heterocycles. One notable limitation with these reactions was the need for high reaction temperatures, typically about 150 °C. In a subsequent study we found that if we used catalysts incorporating P­(*t*-Bu)_2_Me as the ligand then a similar range of reactions could be achieved but at the reduced temperature of 120 °C. These lower temperatures allowed the use of coupling partners featuring functional groups that were not tolerated at 150 °C, for example, phenols, NH-carbamates, and NH-sulfonamides could now be incorporated; Scheme 1b shows selected examples.^33^ In addition, lowering temperatures close to the solvent’s boiling point allowed reactions to be performed in standard sealed vials instead of specialized high-pressure equipment. In this second iteration we also demonstrated that a broader range of heterocyclic sulfinates were useful substrates, with pyrazine, pyridazine, and pyrimidine sulfinates all working well, expanding this methodology to other systems prone to proto-deboronation. At this point we had addressed many of our initial goals, in that we had identified 2-*aza*-aryl sulfinates as effective nucleophilic coupling partners that react well with a broad range of (hetero)­aryl halides. The reactions were efficient, and the sulfinate substrates, which were generally crystalline solids, displayed good stability. The sulfinate reagents could also be prepared from a varied range of precursors, including the corresponding thiols,^34^ and halides.^35–37^ At the outset of our work there were no commercially available pyridine sulfinates, but a recent survey of the SciFinder database returned >450 commercial metal sulfinates featuring the core 2-pyridyl and related motifs.^38^

**Scheme 1:**
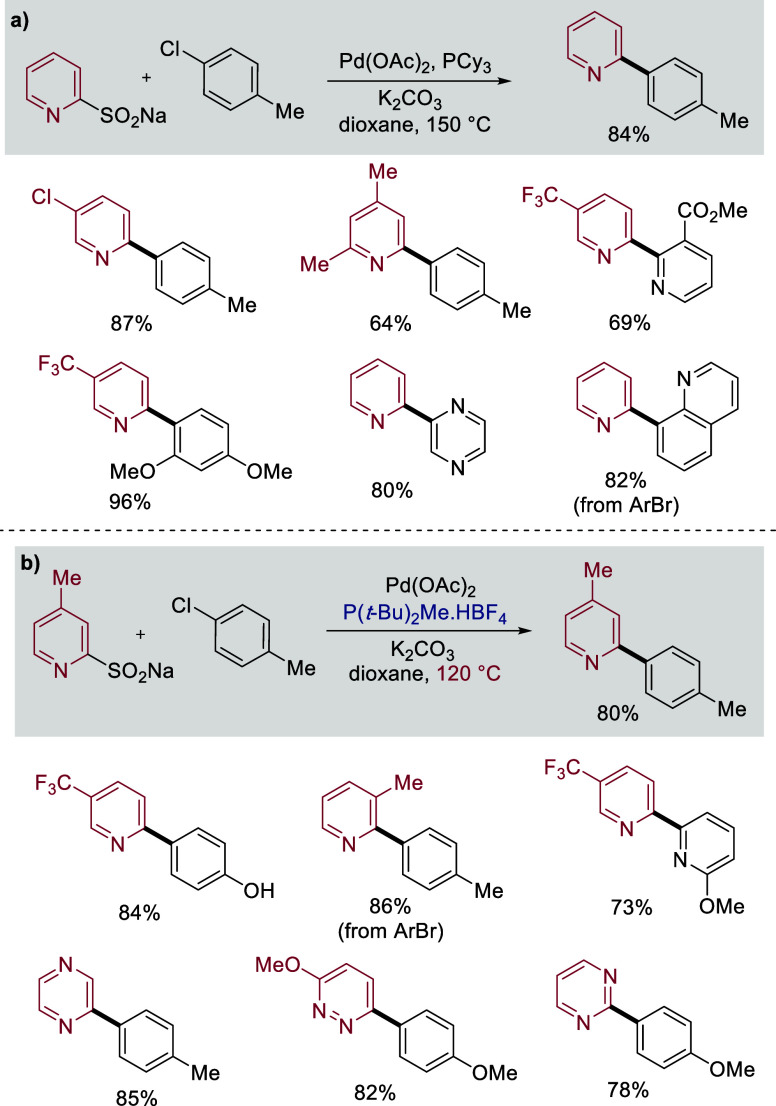
Pyridine Sulfinates in Palladium-Catalyzed Coupling Reactions with Aryl Halides

In a later study we have also explored the mechanism of these reactions in detail.^39^ Among others, a key observation from this study was that the turnover limiting step, for reactions that employ 2-pyridyl sulfinate reagents, was extrusion of SO_2_ from a chelate intermediate formed between the palladium center (after oxidative addition to the aryl halide) and the sulfinate reagent. These chelate complexes were also found to be the resting state intermediates.

Despite the success of the 2-pyridyl sulfinates, the reagents are salts, which sometimes led to challenges with the purification or isolation of more complex sulfinates. To circumvent these issues, we were interested in developing coupling partners that retained the excellent reactivity of the metal sulfinate salts, but which were noncharged organic molecules. Importantly, we did not want to engineer a system that required a separate “deprotection” step to form a sulfinate; the reactive sulfinates should be generated under the standard reaction conditions. Our investigations led us to first develop 2-pyridyl allylsulfones as latent sulfinate reagents.^40^ These allylsulfone reagents are straightforward to prepare, most easily from the corresponding thiol using an allylation/oxidation sequence, with the intermediate sulfide telescoped through to the final sulfone. Direct allylation of the sulfinate salts is also possible, as are S_N_Ar approaches, and in all cases the allylsulfone products were nonproblematic to purify. Mechanistically, these allylsulfone reagents were designed to operate under the Pd(0) conditions used in the sulfinate coupling reactions. The reactions proceed via a Pd(0)-catalyzed fragmentation of the allylsulfone to generate a π-allyl-Pd intermediate, while at the same time releasing the pyridyl sulfinate as a leaving group. Interception of the π-allyl-Pd intermediate with a nucleophile (presumed to be carbonate in these reactions) would regenerate Pd(0) and allow the coupling reaction to proceed.

Cross-coupling reactions using these allylsulfone reagents proceeded under conditions very similar to those used for the parent sulfinates, with the switch of base from K_2_CO_3_ to Cs_2_CO_3_ and the use of DMF instead of 1,4-dioxane as solvent for certain substrates being the only changes. Using these conditions, a broad range of pyridyl allylsulfones were combined efficiently with a wide selection of aryl and heteroaryl halides, with selected examples being shown in Scheme 2a. Importantly, in a series of control reactions we had demonstrated that the allylsulfone fragment was able to withstand a range of synthetic transformations, such as the DIBAL-H reduction of an ester, Fe-mediated nitro reduction, and basic nitrile hydrolysis. Good stability to Chan-Lam arylation, Cu-catalyzed Ullman-type couplings, as well as Pd-catalyzed Buchwald-Hartwig arylation was also demonstrated. These stability studies allowed chemo-selective couplings to be achieved using multifunctional reagents. For example, a standard Suzuki-Miyaura coupling between 3-Br-5-Cl-2-pyridine allylsulfone **1** and sulfonyl boronic acid **2** delivered biaryl **3**, with the aryl chloride and allylsulfone functionalities remaining intact, in an excellent 75% yield (Scheme 2b). Deallylative/desulfonylative coupling between sulfone **3** and 3-Br-6-Me-pyridine then delivered the COX-2 inhibitor etoricoxib in 69% yield.

**Scheme 2:**
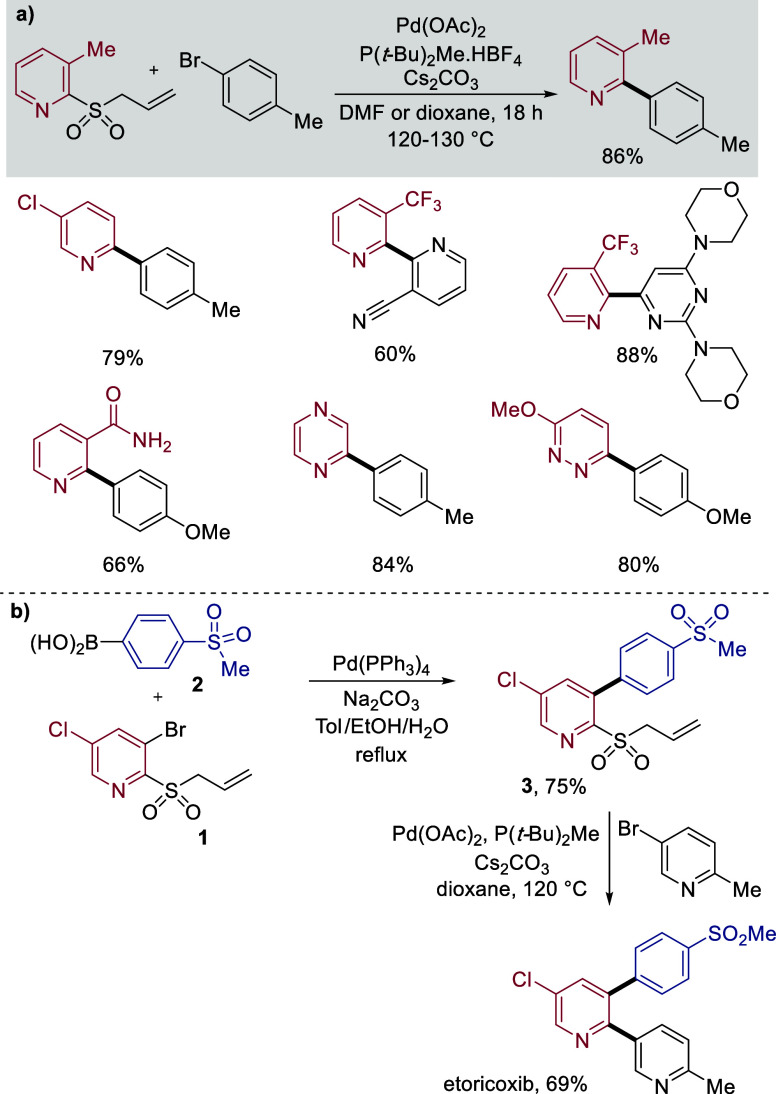
a) Pyridine Allylsulfones in Palladium-Catalyzed Cross-Coupling Reactions; b) Etoricoxib Synthesis from Allylsulfone **1** and Boronic Acid **2**

We have also developed a second class of latent sulfinate reagents, this time focusing on reagents that would be released under the basic conditions used in the cross-coupling reactions. Capitalizing on the advantages achieved with the allylsulfone reagents, we decided to remain with a sulfone group, but to use β-ester and β-nitrile-substituted variants (Scheme 3).^41^ These reagents were expected to provide complementary stability to the allylsulfones, for example, avoiding undesirable Heck coupling, and to tolerate reagents known to react well with alkenes, such as oxidative reagents like *m*-CPBA, or alkene metathesis catalysts. In this case, the metal sulfinates were liberated under the action of mild bases via an E1cB mechanism. These new reagents could be prepared from either: (1) the corresponding thiols and the appropriate Michael acceptors or β-bromoesters followed by oxidation,^42^ or (2) the corresponding pyridyl halides using reagents such as SMOPS,^36,43^ β-thioesters followed by oxidation,^14,18^ or via insertion of SO_2_ followed by conjugate addition.^44^ The reagents were straightforward to purify and displayed good stability.

**Scheme 3:**
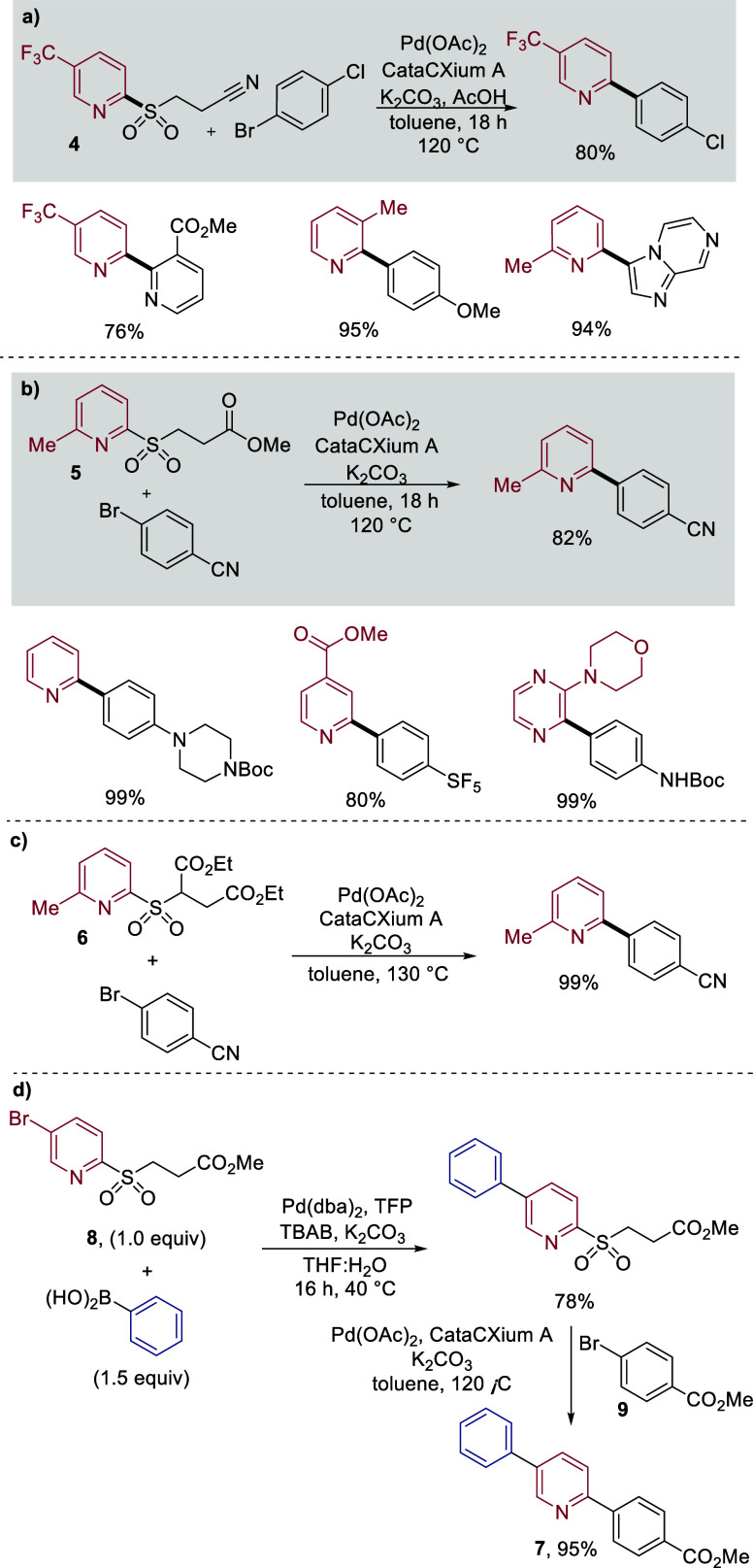
a) Pyridine β-Nitrilesulfones and b) β-Estersulfones in Palladium-Catalyzed Cross-Coupling Reactions; c) Diethyl Succinate Derived Sulfones in Palladium-Catalyzed Cross-Coupling Reactions; d) Synthesis of Diarylpyridine **7** from Bromo Sulfone **8**

The best reaction conditions for the cross-coupling reactions using these β-cyano/ester pyridyl sulfones were slightly different from those reported for the earlier reagents. The ligand cataCXium A [P­(Ad)_2_Bu] was found to be optimal, as was using toluene as solvent. The cyano-reagents (**4**) benefitted from using a combination of K_2_CO_3_ with AcOH as an additive (Scheme 3a), although only K_2_CO_3_ was used for the ester system (**5**, Scheme 3b). The reactions were performed at 120 °C. These sulfones work very well as nucleophilic partners in the cross-coupling reaction, and our original report contains >60 examples using these new reagents. Scheme 3 shows a small selection of coupled products obtained with this method and highlights the functional group tolerance and efficient reactions that can be achieved. We briefly explored the chemistry of a third class of base-labile latent sulfinate reagents, this time selecting to study diethyl succinate derived sulfones **6**. These reagents were of interest because upon generation of the sulfinate salt, they release nontoxic, not prone to spontaneous polymerization, high-boiling diethyl fumarate as the byproduct, making them more amenable to cross-coupling on larger scale. Additionally, the majority of these reagents could be readily isolated as solids. The succinate pyridine sulfones performed well under the same conditions used for the β-sulfonyl methyl esters and delivered cross-coupled products in high yields (Scheme 3c).

To showcase the compatibility of these β-ester/nitrile reagents under alkene functionalization conditions, we demonstrated stability of the sulfones to Wacker oxidation as well as hydroboration/oxidation reaction conditions. If multifunctionalized substrates were used, then bifunctional cross-coupling sequences could also be achieved. For example, diarylpyridine **7** was obtained in high yield from a two-step sequence starting from 5-bromopyridine sulfone **8**, whereby an initial Suzuki–Miyaura cross-coupling with phenyl boronic acid was followed by the desulfinative coupling using ester-substituted aryl bromide **9**.

A very recent publication from Sohn and co-workers reports the synthesis and use of pyridyl 2-pyrimidyl sulfones as latent sulfinate reagents, with the pyridyl sulfinates being released via a S_N_Ar cleavage of S–C bond at the pyrimidine under the action of a mild base but at an elevated temperature.^45^

In addition to building challenging pyridyl-(hetero)­aryl bonds, we have also explored a desulfinative cross-coupling process toward robust, versatile syntheses of diarylmethanes. The aim was to overcome long-standing problems of aromatic benzylation, including: various inefficient multistep approaches, often unreliable Negishi couplings, use of unstable benzyl boronates, and the lack of versatility in emerging Ni-catalyzed benzyl cross-electrophile couplings.^46^ Toward this end, we have developed a reductant free cross-electrophile coupling for the synthesis of diarylmethanes based on the in situ formation of benzylic sulfinates.^47^ The overall transformation, together with selected examples, is shown in Scheme 4, and involves the direct combination of a benzylic halide, a (hetero)­aryl halide, the SMOPS reagent, and a palladium catalyst. The reactions proceed via initial formation of a β-ester substituted benzylic sulfone (**10**) which then undergoes elimination to generate the key benzylic sulfinate (**11**). From **11**, palladium-catalyzed desulfinative coupling delivers the targeted diarylmethanes. The process tolerates a good range of functional groups on both reaction partners.

**Scheme 4:**
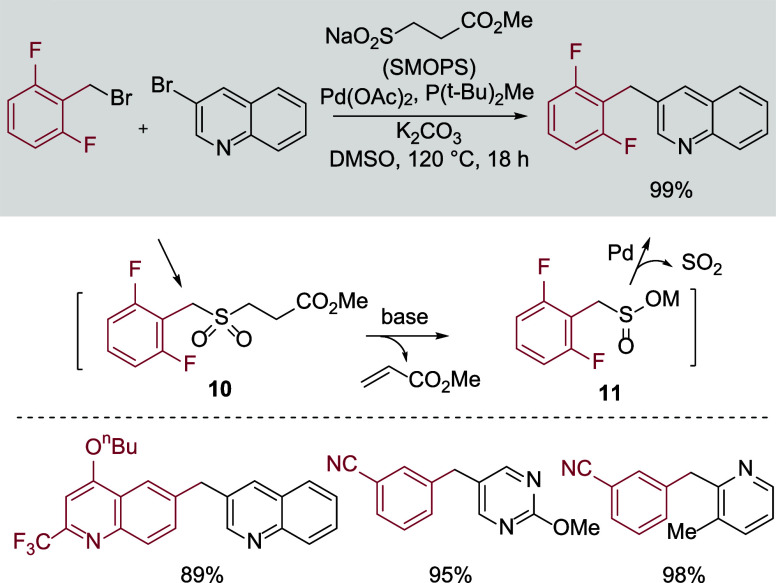
Reductant-Free Cross-Electrophile Synthesis of Diarylmethanes

In our first report on the pyridine sulfinate cross-coupling we described the application of the reagents in two small array experiments, delivering libraries of varenicline and mepyramine derivatives.^32^ We also demonstrated that the reactions could be used to supply gram-scale quantities of coupled products, with a reaction of >6 g scale being successfully achieved. Following our initial reports, the chemistry has been adopted by other chemists, working in both academic and industrial laboratories. An early example of the use of the sulfinate reagents came from Rowlands and co-workers, who used the pyridine sulfinate reagents in the synthesis of chiral pyridyl[2.2]­paracyclophanes.^48^ Of more relevance from a medicinal chemistry perspective, are a series of patents from GSK addressing mTOR kinase inhibition.^49,50^ In these reports they describe several examples of complex pyridine sulfinate reagents coupling with complex heteroaryl halides. Scheme 5a shows one example, in which >28 g of bipyridine **12** is obtained in 79% yield from the union of pyridine sulfinate **13** and chloro azaindole **14**. The reactions are achieved using the original PCy_3_-derived catalyst we reported. A series of patents from Ildong Pharma,^51,52^ starting in 2021, employ a range of 2-pyridine sulfinate reagents in coupling reactions with chloro-pyrazolopyrimidine derivatives (**15**), in a program toward developing A2A/A1 antagonists (Scheme 5b). PCy_3_-derived catalysts were again used. Janssen have exploited the sulfinate reagents in work toward antibacterial compounds;^53^ an example coupling, using a chloro-triazolopyrazine coupling partner (**16**) is shown in Scheme 5c.

**Scheme 5:**
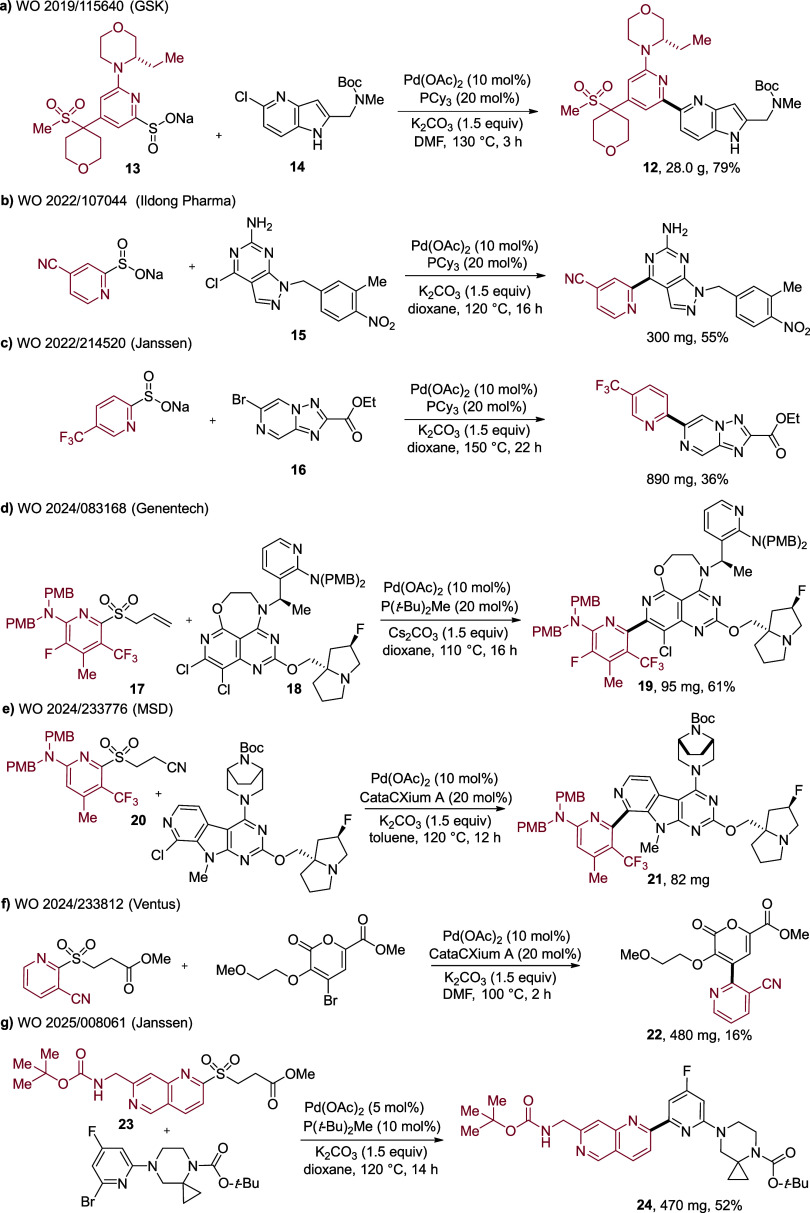
(a–g) Applications of Palladium-Catalyzed Desulfinative Cross-Couplings for the Synthesis of 2-Pyridyl Derivatives

The masked sulfinate reagents have also been applied in medicinal chemistry laboratories. In a program targeting KRas, Genentech were able to couple penta-substituted pyridyl allylsulfones such as **17** with complex chloro-pyridopyrimidines such as **18** to form bipyridine **19** in respectable yield (Scheme 5d).^54,55^ These couplings employed the P­(*t*-Bu)_2_Me-derived catalyst, were conducted at 110 °C, and provided some of the largest molecules prepared using desulfinative couplings. In addition, KRas chemistry from MSD has utilized β-nitrile sulfones such as **20** in a range of coupling reactions; Scheme 5e shows an example in which linked pyridine **21**, a large molecule containing multiple nitrogen atoms, is prepared using the CataCXium-derived catalyst.^56^ β-Estersulfone coupling partners were also employed by Ventus to prepare pyridine-substituted pyranones such as **22** in moderate yields. The CataCXium-derived catalyst was used in these reactions, and reaction times of only 2 h were used. These reduced reaction times may suggest product or substrate stability issues (Scheme 5f).^57^ The final example, as shown in Scheme 5g, demonstrates an application from Janssen. In this chemistry, 1,6-naphthyridine-derived β-estersulfones such as **23** were combined with bromo-pyridine coupling partners to form bipyridine-like structures such as **24**, this time using the P­(*t*-Bu)_2_Me-derived catalyst.^58,59^

In recent decades, parallel medicinal chemistry (PMC) has become an essential tool in modern drug discovery, allowing for the rapid, efficient, and simultaneous synthesis of many compounds by conducting the same bond-forming reaction typically between one constant scaffold (template) and a variety of coupling partners introducing structural diversity. The impact of a PMC protocol is determined by versatility, robustness, and good functional group tolerance of the key synthetic transformation.^60^ While generally meeting these criteria, due to its demonstrated effectiveness and coverage of difficult-to-access chemical space, the desulfinative coupling has shown the potential to become a standard and productive technique for lead optimization through broader chemical diversification of synthetic libraries. Medicinal chemists at Pfizer have successfully used desulfinative coupling in the preparation of small-, medium-, and large-size libraries as part of PMC-driven strategies in internal drug discovery programs. Some unpublished examples of template synthesis and key bond-forming reactions are presented in Schemes 6 and 7,^61^ with varying fragments (and corresponding building blocks) highlighted in blue; the number of compounds successfully synthesized using PMC protocols and success rates are provided.^62^ The value of this methodology is again confirmed by the fact that in all shown here and numerous other unpublished examples, it provided solutions where Suzuki, Negishi, and other types of alternative conditions were either unproductive or unpractical.

**Scheme 6:**
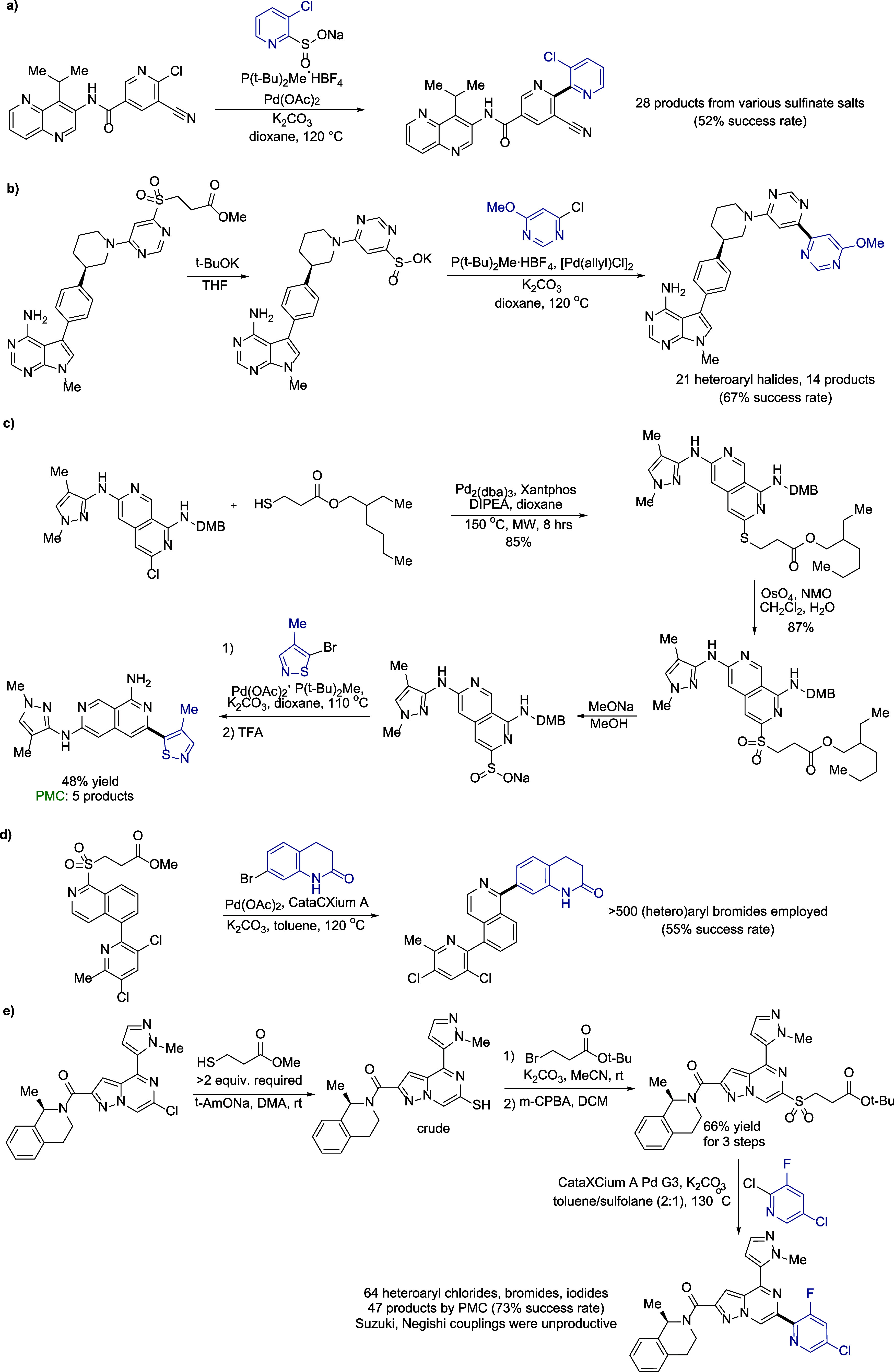
Parallel Medicinal Chemistry Applications of Desulfinative Cross-Coupling (Unpublished Data)

**Scheme 7:**
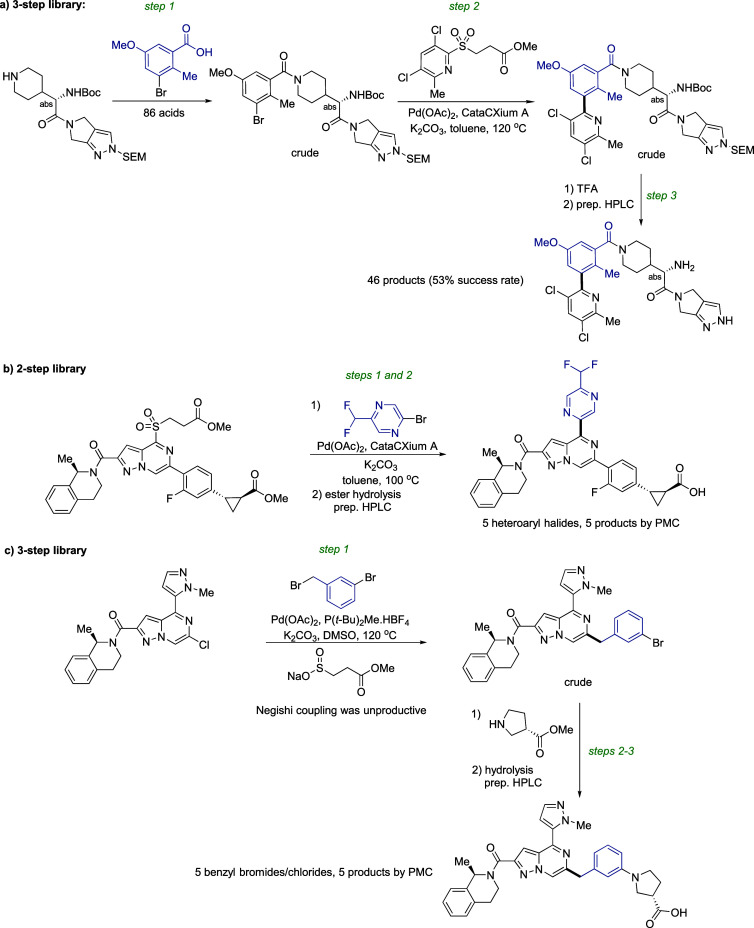
Parallel Medicinal Chemistry Applications of Desulfinative Cross-Coupling (Unpublished Data)

Scheme 6a showcases a productive 2,2’-bipyridyl synthesis with both coupling partners carrying *ortho*-substituents. The bipyridyl fragment was critical in the series and desulfinative coupling was the only way to build it reliably as the corresponding boronates were unstable. Scheme 6b illustrates an instance when the sulfinate salt was preferred as the coupling substrate over its sulfonyl ester precursor because the latter was susceptible to C–S bond hydrolytic cleavage under the reaction conditions. A nonodorous commercially available β-thioester was used in the preparation of a sulfinate template in Scheme 6c; the unsaturated ester byproduct in this case is also less toxic and less volatile than typically generated methyl acrylate, which is beneficial in scale-up syntheses. In Scheme 6d a PMC application was achieved with extensive variation of the aryl bromide coupling. Scheme 6e includes some additional findings useful in the field: 1) thiolation of a chloroheterocycle was successfully carried out using β-thioester (with in situ β-elimination) where thiolation with thiourea or NaSH was unproductive; 2) if equally accessible, *tert*-butyl sulfonyl esters usually give higher yields in desulfinative coupling than methyl esters due to better hydrolytic stability under the reaction conditions.^20^ The starting chloro pyrazolopyrazine was unreactive toward aryl boronates or aryl zincates, making desulfinative coupling indispensable.

Schemes 6c and 7a–c show examples of multistep PMC, where intermediates are carried to the next step without purification. In all the examples of Schemes 6 and 7, desulfinative coupling proved to be the most productive and uniquely effective way to achieve the required structural diversity and synthesis speed, with minimal optimization efforts in each case. Importantly, after PMC experiments, the identified hits were scaled up to gram or higher quantities in some of the examples presented here and other proprietary series. In most cases, desulfinative coupling was found to have good reproducibility on larger scale.

In conclusion, we have developed a series of 2-*aza-*aryl reagents suitable for efficient and reliable palladium-catalyzed couplings with a broad range (hetero)­aryl halides. The choice of the 2-*aza-*aryl reagent in a particular reaction (sulfinate salt, or a related “masked″ sulfinate reagent) depends on several factors: the relative stability and reactivity of coupling partners at the reaction temperature, the reaction scale, the complexity and accessibility of the substrate(s), and the synthetic strategy adopted for a specific final target (i.e. whether the reagent is introduced early or late in the sequence). The nature of the 2-*aza-*aryl reagent dictates the most appropriate catalyst choice, although some flexibility exists; high-value syntheses benefit from further optimization of the ligand, palladium source, solvent, and reaction temperature. From the examples presented from our own laboratories, and those of medicinal and synthetic chemists around the world, it is clear that desulfinative couplings of 2-*aza-*aryl reagents can be used to prepare complex, multi-functionalized molecules, and that the chemistry can be performed on scales ranging from milligrams to tens of grams. Although there remain limitations, most notably the relatively high reaction temperatures needed, the presented chemistry provides a solution to many of the challenges associated with 2-*aza-*aryl boronates.

## Abbreviations

cataCXium A: P­(Ad)_2_Bu
DIBAL-H: diisobutylaluminum hydride
*m*-CPBA: *meta*-chloroperbenzoic acid
PMC: parallel medicinal chemistry
SMOPS: sodium 1-methyl 3-sulfinopropanoate
TFP: tri­(2-furyl)­phosphine
